# IPGA: A handy integrated prokaryotes genome and pan‐genome analysis web service

**DOI:** 10.1002/imt2.55

**Published:** 2022-09-14

**Authors:** Dongmei Liu, Yifei Zhang, Guomei Fan, Dingzhong Sun, Xingjiao Zhang, Zhengfei Yu, Jinfeng Wang, Linhuan Wu, Wenyu Shi, Juncai Ma

**Affiliations:** ^1^ Microbial Resource and Big Data Center, Institute of Microbiology Chinese Academy of Sciences Beijing China; ^2^ Central Laboratory Peking University School and Hospital of Stomatology Beijing China; ^3^ College of Food Science and Nutritional Engineering China Agricultural University Beijing China; ^4^ State Key Laboratory of Microbial Resources, Institute of Microbiology Chinese Academy of Sciences Beijing China; ^5^ National Microbiology Data Center Beijing China

**Keywords:** comparative genomics, pan‐genome, web service

## Abstract

Pan‐genomics is one of the most powerful means to study genomic variation and obtain a sketch of genes within a defined clade of species. Though there are a lot of computational tools to achieve this, an integrated framework to evaluate their performance and offer the best choice to users has never been achieved. To ease the process of large‐scale prokaryotic genome analysis, we introduce Integrated Prokaryotes Genome and pan‐genome Analysis (IPGA), a one‐stop web service to analyze, compare, and visualize pan‐genome as well as individual genomes, that rids users of installing any specific tools. IPGA features a scoring system that helps users to evaluate the reliability of pan‐genome profiles generated by different packages. Thus, IPGA can help users ascertain the profiling method that is most suitable for their data set for the following analysis. In addition, IPGA integrates several downstream comparative analysis and genome analysis modules to make users achieve diverse targets.

## Highlights

- IPGA serves as a free and easy‐to‐use web‐based system that could provide up‐to‐date pan‐genome analysis service for non‐bioinformaticians.
- IPGA offers users the most reliable pan‐genome profile which enables users to perform additional comparative genomic analysis.
- IPGA provides a series of downstream analysis modules such as phylogenetic inference, synteny inference, and target genome annotation.

## Graphical abstract

Integrated Prokaryotes Genome and pan‐genome Analysis (IPGA) serves as a free and easy‐to‐use web‐based system that could provide up‐to‐date pan‐genome analysis service for non‐bioinformaticians. IPGA offers users the most reliable pan‐genome profile which enables users to perform additional comparative genomic analysis. IPGA provides a series of downstream analysis modules such as phylogenetic inference, synteny inference, and target genome annotation.

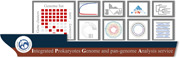

## INTRODUCTION

Pan‐genome analysis, being one of the most important approaches used in comparative genomics, has been widely used in the study of the diversity and evolutionary relationships of prokaryotes, the prevention and control of infectious disease, and the surveillance of drug resistance among pathogens [1, 2]. The aim of the pan‐genome analysis is to evaluate the variability of all genes and genomic structures from genomes within a certain clade. The initial and most important step in the pan‐genome analysis is the clustering of orthologous genes. Gene clusters are then divided into three groups according to their occurrence in the given sets of genomes: core genes, accessory genes, and unique genes. Currently, there are a number of packages or web services [3, 4, 5, 6, 7, 8, 9, 10, 11, 12] (Supporting Information: Table S1) that can achieve this goal, but the results of pan‐genome profiling could end up very differently. Therefore, an evaluation process should be involved to estimate the reliability of these profiles. Besides that, the downstream analysis and visualization of the target strains of sub‐clades can also constrain the study of the pan‐genome of prokaryotes for non‐bioinformaticians.

Here we present IPGA, a powerful and practical online service to facilitate the analysis of pan‐genome and genomes of chosen clades. IPGA integrates 8 pan‐genome analysis packages and provides an evaluation strategy to help users select the best pan‐genome profile. In addition, IPGA enables users to perform downstream analysis workflows, such as phylogenetic inference, synteny inference, and target genome annotation at the same time.

## IMPLEMENTATION

IPGA was developed based on National Microbiology Data Center (NMDC) cloud system, which provided adequate computing resources to ensure IPGA's services. The cloud dynamic scheduling system was developed based on Mesos and Marathon frameworks. All the packages in IPGA were individually dockerized and launched by the scheduling system. The website front was developed by spring cloud framework with Java SE Development Kit 8. In addition, IPGA was supported by a systematically curated prokaryotes genome database in NMDC through a data portal API. So that, users could simply provide genome accession IDs for the publicly available genomes instead of uploading the genome files.

**Figure 1:**
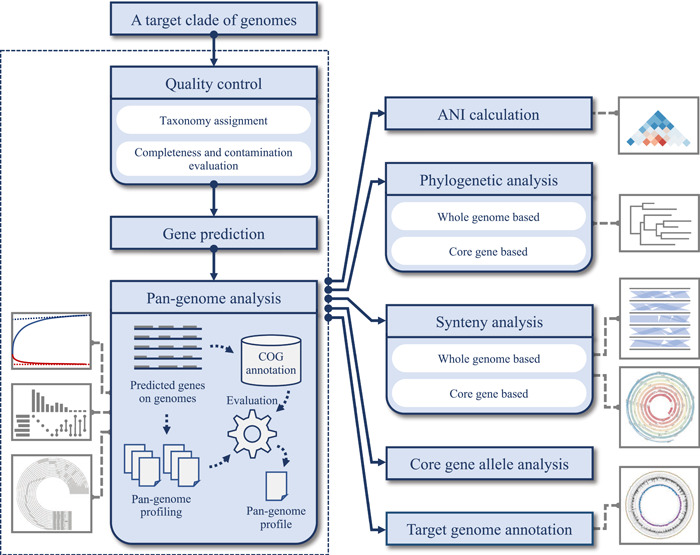
Workflow of IPGA. IPGA, Integrated Prokaryotes Genome and pan‐genome Analysis.

IPGA workflow accepts a set of prokaryotic genomes as input. Then, 7 analysis modules can be selected to start the IPGA workflow (Figure 1 and Supporting Information: Figure S1). The quality control module is initial in IPGA, which is used to remove all low‐quality genomes and perform a taxonomic assignment for each genome. IPGA then predicts genes of all filtered genomes and uses them as the input of the pan‐genome analysis module. After pan‐genome profiles were created by different software, IPGA extracts all links between every pair of genes that belong to the same core gene cluster, and then, counts the number of shared links between different pan‐genome profiles annotated by different software. IPGA scores every profile using the following strategy (Supporting Information: Figure S2):

**Table:** 

**FOR** each gene_i_ and gene_j_:
**IF** gene_i_ and gene_j_ can be both annotated in COG database [13]:
**FOR** each pan‐genome profile_s_:
**IF** gene_i_ and gene_j_ have same COG annotation **AND**
gene_i_ and gene_j_ are grouped in the same cluster in pan‐genome profile_s_:
score_s_++
**IF** gene_i_ and gene_j_ have different COG annotation **AND**
gene_i_ and gene_j_ are separated into the different cluster in pan‐genome profile_s_:
score_s_++
**ELSE**:
**FOR** each pan‐genome profile_s_:
**IF** gene_i_ and gene_j_ are in the same cluster in more than *x* pan‐genome profiles **AND**
gene_i_ and gene_j_ are grouped in the same cluster in pan‐genome profile_s_:
score_s_++
**IF** gene_i_ and gene_j_ are in the different cluster in less than *x* pan‐genome profiles **AND**
gene_i_ and gene_j_ are separated into the different cluster in pan‐genome profile_s_:
score_s_++

In the pseudo codes, gene_i_ and gene_j_ are a pair of genes that are grouped in the same cluster in any pan‐genome profile; s represents the pan‐genome profile created by the software *s*; the integer cut‐off value *x* represents that there are *x* pan‐genome profiles reporting gene_i_ and gene_j_ in the same cluster; score_s_ represents the score of the pan‐genome profile created by the software *s*. The profile with the highest score will be reported. After that, filtered genomes and gene clusters will be used in the next four downstream comparative genomic analysis modules including the phylogenetic analysis module, synteny inference module, core gene allele analysis, and average nucleotide identity (ANI) calculation module. In addition, a list of genomes can be selected to be annotated in the genome annotation module. Any module can be skipped if it is not the prerequisite for the selected workflow. After all input genome sequences are uploaded and all parameters are determined, the IPGA workflow can be started. Job status and the results would be sent to the job submitter via e‐mail.

## RESULTS

### The performance of the pan‐genome analysis module in IPGA on different data sets

**Figure 2:**
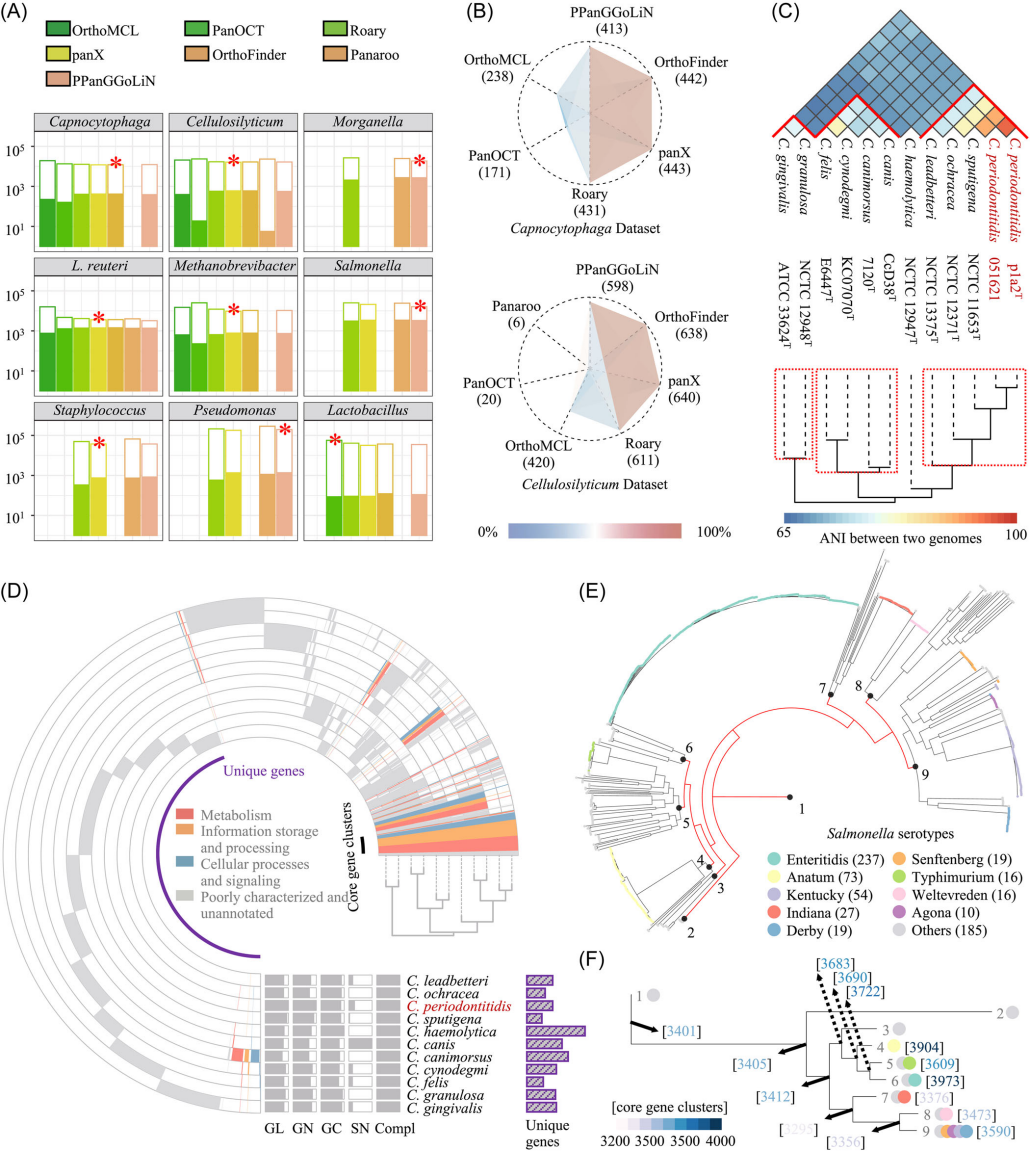
Results of IPGA on different data sets. (A) The performance evaluation on nine different data sets. The height of the bars represents the number of total orthologous gene clusters estimated by each tool, and the colored part represents the number of core genes predicted by each tool. Tools are color‐coded, and the best tool for each data set is marked by an asterisk. (B) The number of shared core gene links among four different softwares. (C) ANI analysis result on data set *Capnocytophaga*. (D) The pan‐genome profile of all type strains in genus *Capnocytophaga*. “GL,” “GN,” “GC,” “SN,” and “Compl” represent genome length, gene number, GC content, scaffold number, and genome completeness, respectively. (E) The phylogenetic inference based on the whole genome variation of data set *Salmonella*. (F) The core gene clusters of nine major cluster of data set *Salmonella*. ANI, average nucleotide identity; IPGA, Integrated Prokaryotes Genome and pan‐genome Analysis.

We demonstrated the performance of the IPGA pan‐genome analysis module using nine different data sets, including metagenome‐assembled genomes, genomes of food‐borne pathogens, and genomes from several bacterial clades. Pan‐genome profiling results of eight software differed a lot among these test data sets (Figure 2A). For example, in the pan‐genome profiles of data set *Capnocytophaga* and *Cellulosilyticum*, IPGA extracted gene pairs grouped in the same clusters, and then, counted the numbers of the same gene pairs between different pan‐genome profiles. Roary, panX, OrthoFinder, and PPanGGoLiN shared the largest amount of links in common (Figure 2B), which were reflected by their high scores calculated by IPGA (Supporting Information: Table S2). Based on the scoring system, IPGA evaluated the best‐performed pan‐genome profiling result. PanOCT, PanX, OrthoFinder, PPanGGoLiN achieved the best in different data sets. Moreover, according to the result of the evaluation process on all data sets, the performance of OrthoFinder, panX, and PPanGGoLiN was relatively stable and Roary underestimated the number of core gene clusters in all data sets, especially in large data sets (>200 genomes). Among all 8 software, Roary and PPanGGOLiN created pan‐genome profiles with fewer computing resources, especially when dealing with data sets that contain more than 500 genomes, whereas some of the software would fail after 2 days (Supporting Information: Figure S3). Compared with the other pan‐genome analysis platform, IPGA offers a performance evaluation and provides a measured pan‐genome profile to perform downstream analysis.

### Case study I: Pan‐genome profiling

The pan‐genome analysis is recommended in the proposal of new species. It could provide genotypic differences as a complement to phenotypic differences between proposed and related strains. We demonstrated a case study using data set *Capnocytophaga*, which contained 12 genomes belonging to 11 species. Among these genomes, *C. periodontitidis* was newly proposed as a type strain [14]. All 11 species in this genus could be divided into four groups based on the value of ANI (cutoff = 75, Figure 2C). Furthermore, the hierarchical structure of the ANI result and pan‐genome profile were identical to the polygenetic tree created based on conserved single‐copy genes (Supporting Information: Figure S4). In 11,716 orthologous gene clusters, only 419 (3.57%) were the core gene clusters, while up to 6976 (59.54%) unique genes belonged to different genomes. Among them, there were more unique genes that belonged to *C. periodontitidis* p1a2^T^ (559) than that belonged to *C. ochracea* (398), *C. sputigena* (273), and *C. felis* (356) (Figure 2D). These results all provided gene‐level evidence indicating that *C. periodontitidis* is highly divergent from the other species in the genus *Capnocytophaga*.

### Case study II: Genome annotation and visualization

A circular genome map [15] is a commonly used case in the illustration of genome annotation. However, using circular genome representation to visualize the fragmented genome, especially for the high‐qualified assembled genomes from metagenome data, is misleading. We demonstrated two genomes in the genus *Cellulosilyticum*, one from an isolated strain (strain WCF‐2) with CGView [16] and a metagenome‐assembled genome (MAG SIG270) with a spiral plot using IPGA (Supporting Information: Figure S5). GC‐skew, GC‐content, and annotation of all genes of two strands were displayed in different rings or lanes.

### Case study III: Phylogenetic inference

IPGA could also help user to perform downstream analysis in the study of a large number of genomes. Six hundred and sixty‐seven high‐quality *Salmonella* genomes, which were isolated from China [17], were used as an example. These genomes could be grouped into nine different clusters (Figure 2E) based on whole‐genome SNP‐based phylogenetic inference. An extremely long branch consisting of 12 genomes was removed, because of the low ANI value (below 97) between these 12 genomes and the others (Supporting Information: Figure S6). The pan‐genome profile was shown in Figure 2F. Thus, users could focus on the gene difference or single nucleotide difference of target clusters based on the detailed output. In addition to the standard phylogenetic analysis, we demonstrated another example using 131 complete genomes of the genus *Lactobacillus*. Users could zoom in on a branch to find out about the synteny between eight genomes in a subclade, seven *L. iners* genomes, and an out‐group genome (Supporting Information: Figure S7).

## CONCLUSION AND FUTURE DIRECTION

Compared with other pan‐genome analysis protocols, IPGA is not only able to greatly improve the reliability of the analysis by selecting the best‐performed result but is also able to provide some further insight into genomic differences between target prokaryotic genomes. The downstream functions integrated into IPGA allow users to finish a complete workflow of regular genomic analysis in a one‐stop‐shop style. Hence, IPGA could be a useful tool for researchers studying pan‐genomics, comparative genomics, and related fields.

IPGA is developed with a modular rationale in mind so as to support easy expansion in the future. Any such future function and upgrade can be found in the “Updates” on the website https://nmdc.cn/ipga/.

## AUTHOR CONTRIBUTIONS

Linhuan Wu, Wenyu Shi, and Juncai Ma conceived the idea. Dongmei Liu and Wenyu Shi designed and developed the workflow. Guomei Fan, Xingjiao Zhang, Zhengfei Yu, and Wenyu Shi established the web service system. Dongmei Liu, Yifei Zhang, Zhengfei Yu, and Wenyu Shi tested the workflow and provided the sample data. Dingzhong Sun, Jinfeng Wang, and Wenyu Shi wrote and corrected the manuscript.

## CONFLICT OF INTEREST

The authors declare no conflict of interest.

## Supporting information

Supporting information.

Supporting information.

## Data Availability

There are no access restrictions for the academic use of IPGA. The main scripts involved in IPGA are available on the “Download” tabpage. All publicly available genomic data or metadata are freely accessible (Data set descriptions in Supplementary tables). Supplementary materials (figures, tables, scripts, graphical abstract, slides, videos, Chinese translated version, and update materials) may be found in the online DOI or iMeta Science http://www.imeta.science/.
