# Supplementary material for: IPGA: A handy integrated prokaryotes genome and pan‐genome analysis web service
Source: Imeta. 2022 Sep 14;1(4):e55. doi: 10.1002/imt2.55 (PMC10989949; doi:10.1002/imt2.55)
Supplement: Supplementary file 1 — Supporting information. [file IMT2-1-e55-s002.docx]

# IPGA: a handy integrated prokaryotes genome and pan-genome analysis web service

Dongmei Liu^1^, Yifei Zhang^2^, Guomei Fan^1^, Dingzhong Sun^1^, Xingjiao Zhang^1^, Zhengfei Yu^1^, Jinfeng Wang^3^, Linhuan Wu^1,4,5^, Wenyu Shi^1*^, Juncai Ma^1,4,5*^

1 Microbial Resource and Big Data Center, Institute of Microbiology, Chinese Academy of Sciences, Beijing 100101, China

2 Central Laboratory, Peking University School and Hospital of Stomatology, Beijing 100081, China

3 College of Food Science and Nutritional Engineering, China Agricultural University, Beijing 100083, China

4 State Key Laboratory of Microbial Resources, Institute of Microbiology, Chinese Academy of Sciences, Beijing 100101, China

5 National Microbiology Data Center, Beijing 100101, China

^*^ To whom correspondence should be addressed: Juncai Ma [ma@im.ac.cn] and Wenyu Shi [shiwy@im.ac.cn].

# SUPPLEMENTARY METHODS

**IPGA Workflow**

IPGA includes 7 analysis modules and accepts a set of genomes as input. Quality control module is the initial step in IPGA workflow. Quality control module integrates checkM [[1](#_ENREF_1)] and GUNC [[2](#_ENREF_2)] to check the completeness and contamination of the input genomes. IPGA will reduce the potential errors during the execution of downstream modules, but it can still be skipped. Besides that, quality control module integrates GTDB-tk [[3](#_ENREF_3)] to perform taxonomic assignment for all input genomes. Next, genes of all filtered genomes were predicted. They would be used in pan-genome analysis module.

In pan-genome analysis module, all genes are annotated against COG database [[4](#_ENREF_4)] and used to create different pan-genome profiles using up to 8 types of software (OrthoMCL [[5](#_ENREF_5)], PanOCT [[6](#_ENREF_6)], Roary [[7](#_ENREF_7)], OrthoFinder [[8](#_ENREF_8)], panX [[9](#_ENREF_9)], Panaroo [[10](#_ENREF_10)], PPanGGoLiN [[11](#_ENREF_11)] and PEPPAN [[12](#_ENREF_12)]). IPGA then provides a score for each of them to help users select the best pan-genome profile from the potentially various results (Figure S2A). IPGA extracts all orthologous gene pairs that were reported in all pan-genome profiles. And then, for an orthologous gene pair generated by each software, the scoring system can be described as (Figure S2B and S2C): (i) if these two genes can be both annotated in the COG database, the score will increase when these two genes obtain the same COG annotation; (ii) if either gene can be annotated in the COG database, then the score increases when more than *x* softwares group the genes or less than *x* softwares ungroup the genes, where *x* is the cutoff value set by users. In IPGA, the default value of *x* equal to the half number of softwares. The profiling result with highest score will be recommended. Noticeably, PEPPAN changes IDs of all the genes and places the same gene into different ortholog clusters. As a result, the output of PEPPAN cannot be used to score in IPGA system.

After obtaining the recommended pan-genome profile, all single copied core genes are aligned using MAFFT [[13](#_ENREF_13)] and then used to estimate phylogenies using FastTree [[14](#_ENREF_14)]. Whole genome based phylogenetic analysis is also performed using kSNP [[15](#_ENREF_15)].

Gene-based syntenic analysis is performed based on the selected pan-genome profile. Conserved gene orders will be reported to users. Genome-based syntenic analysis is performed using MUMmer [[16](#_ENREF_16)]. Single base variations on core genes are extracted from MUMmer output and then reported to users.

For genome analysis module, ANI values between each submitted genome pairs are calculated. Represented genome set will be reported to users based on the ANI results. Then, IPGA will perform genome annotation of each of genomes described in gcType [[17](#_ENREF_17)] in given target genome list.

Detailed parameters can be seen in Table S4.

The main script involved in IPGA are available in the 'Download' tabpage in https://nmdc.cn/ipga/.

**Mock datasets and the performance**

Mock datasets are created using SimPan (https://github.com/zheminzhou/SimPan), following the instructions described on the Github site and in the supplementary material (Supplemental Text2 and Supplemental Table S1) of reference [[12](#_ENREF_12)]. We have tested the performance of IPGA with 15 mock datasets, and its results are consistent, with the highest scored package giving the closest estimates to the numbers of core genes.

**Test datasets**

In this study, 9 different datasets were used to test the performance of IPGA (Dataset descriptions in Supplementary tables):

1. ***Capnocytophaga* dataset** contains 12 genomes that cover all the validly published type strains of genus *Capnocytophaga* [[18](#_ENREF_18)];

2. ***Cellulosilyticum* dataset** contains 8 genomes that cover the genus *Cellulosilyticum*. *Cellulosilyticum* had been widely reported in the microbiota studies of digestive system of large mammals;

3. ***Morganella* dataset** contains 202 genomes from genus *Morganella*, and all of them belong to species *M. morganii*, an opportunistic pathogen in intestinal tracts;

4. ***L. reuteri* dataset** contains 16 genomes, which were isolated and sequenced from the feces of different hosts, were published in previous study [[19](#_ENREF_19)];

5. ***Methanobrevibacter* dataset** contains 25 genomes collected from NCBI and the study of gastrointestinal tract microbiomes in ruminants [[20](#_ENREF_20)];

6. ***Salmonella* dataset** contains 667 genomes that cover all *Salmonella* genomes (N50>100Kbps) isolated from China based on the record of Enterobase [[21](#_ENREF_21)];

7-9. ***Staphylococcus* dataset**, ***Pseudomonas* dataset** and ***Lactobacillus* dataset** contains 1252, 1231, 831 and 131 complete genomes from each genus, respectively.

# SUPPLEMENTARY FIGURE LEGENDS

**Figure S1. Web interface of IPGA.** There are two steps in a job submission: the first step is to select, upload, and then, confirm the input genome sequences; the second step is to select the analysis modules, analysis parameters and provide the e-mail address to receive the output link.

**Figure S2. Pan-genome analysis module and the scoring system in IPGA for selecting the best pan-genome profile. (A)** A sketch workflow of pan-genome analysis module in IPGA. (**B)** A schema of a typical pan-genome profile. Each dashed region refers to an orthologous gene cluster predicted by pan-genome analysis software. Gene_i,j_ represents the *j*-th genes in genome_i_. S_i_ represents the *i*-th pan-genome analysis software. **(C)** A schema of how IPGA score orthologous gene pairs in different pan-genome profiles generated by pan-genome software. A tick mark means there is a corresponding orthologous gene pair existed, and a cross means there is not. Red color represents an increase of score of the corresponding pan-genome profile. Green color represents no such increase.

**Figure S3. Time consumption of different pan-genome analysis software in IPGA on 9 datasets.**

**Figure S4. hierarchical cluster of pan-genome profile on dataset *Capnocytophaga*.** The numbers close to strain ID represent the number of orthologous gene clusters. The colored numbers on the left side in the parentheses indicate the shared gene clusters, and the black numbers inside indicate the total of non-redundant gene clusters of the pair.

**Figure S5. Genome annotations of two isolates from dataset *Cellulosilyticum*.** **(A)** CGView plot shows GC skew, GC content, and the coding and non-coding gene on the two strands, from center to the outside, respectively. **(B)** Spiral plot on right shows the GC skew, and the coding gene on the two strands, from center to the outside, respectively.

**Figure S6. Removed 11 genomes in dataset *Salmonella*.** The genomes in red frame are removed. **(A)** ANI analysis result of removed 11 genomes and other random selected 66 genomes in dataset *Salmonella*. **(B)** The phylogenetic inference based on the whole genome variation of dataset *Salmonella*.

**Figure S7.** **The phylogenetic tree based on the whole genome variation of dataset Lactobacillus.** The enlarged area shows the synteny visualization of a sub-clade on genome level. Pan-genome statistic visualization is showed on the top-right.

# REFERENCES

1. Parks, Donovan H., Michael Imelfort, Connor T. Skennerton, Philip Hugenholtz, Gene W. Tyson. 2015. “CheckM: assessing the quality of microbial genomes recovered from isolates, single cells, and metagenomes.” *Genome Res* 25: 1043-1055. https://doi.org/10.1101/gr.186072.114

2. Orakov, Askarbek, Anthony Fullam, Luis Pedro Coelho, Supriya Khedkar, Damian Szklarczyk, Daniel R. Mende, Thomas S. B. Schmidt, Peer Bork. 2021. “GUNC: detection of chimerism and contamination in prokaryotic genomes.” *Genome Biology* 22: 178. https://doi.org/10.1186/s13059-021-02393-0

3. Chaumeil, Pierre-Alain, Aaron J. Mussig, Philip Hugenholtz, Donovan H. Parks. 2020. “GTDB-Tk: a toolkit to classify genomes with the Genome Taxonomy Database.” *Bioinformatics* 36: 1925-1927. https://doi.org/10.1093/bioinformatics/btz848

4. Galperin, Michael Y., Yuri I. Wolf, Kira S. Makarova, Roberto Vera Alvarez, David Landsman, Eugene V. Koonin. 2021. “COG database update: focus on microbial diversity, model organisms, and widespread pathogens.” *Nucleic Acids Research* 49: D274-D281. https://doi.org/10.1093/nar/gkaa1018

5. Li, Li, Christian J. Stoeckert, Jr., David S. Roos. 2003. “OrthoMCL: identification of ortholog groups for eukaryotic genomes.” *Genome Res* 13: 2178-2189. https://doi.org/10.1101/gr.1224503

6. Fouts, Derrick E., Lauren Brinkac, Erin Beck, Jason Inman, Granger Sutton. 2012. “PanOCT: automated clustering of orthologs using conserved gene neighborhood for pan-genomic analysis of bacterial strains and closely related species.” *Nucleic Acids Research* 40: e172-e172. https://doi.org/10.1093/nar/gks757

7. Page, Andrew J., Carla A. Cummins, Martin Hunt, Vanessa K. Wong, Sandra Reuter, Matthew T. G. Holden, Maria Fookes, Daniel Falush, Jacqueline A. Keane, Julian Parkhill. 2015. “Roary: rapid large-scale prokaryote pan genome analysis.” *Bioinformatics* 31: 3691-3693. https://doi.org/10.1093/bioinformatics/btv421

8. Emms, David M., Steven Kelly. 2019. “OrthoFinder: phylogenetic orthology inference for comparative genomics.” *Genome Biology* 20: 238. https://doi.org/10.1186/s13059-019-1832-y

9. Ding, Wei, Franz Baumdicker, Richard A. Neher. 2018. “panX: pan-genome analysis and exploration.” *Nucleic Acids Research* 46: e5-e5. https://doi.org/10.1093/nar/gkx977

10. Tonkin-Hill, Gerry, Neil MacAlasdair, Christopher Ruis, Aaron Weimann, Gal Horesh, John A. Lees, Rebecca A. Gladstone, et al. 2020. “Producing polished prokaryotic pangenomes with the Panaroo pipeline.” *Genome Biology* 21: 180. https://doi.org/10.1186/s13059-020-02090-4

11. Gautreau, Guillaume, Adelme Bazin, Mathieu Gachet, Rémi Planel, Laura Burlot, Mathieu Dubois, Amandine Perrin, et al. 2020. “PPanGGOLiN: Depicting microbial diversity via a partitioned pangenome graph.” *PLoS Comput Biol* 16: e1007732. https://doi.org/10.1371/journal.pcbi.1007732

12. Zhou, Zhemin, Jane Charlesworth, Mark Achtman. 2020. “Accurate reconstruction of bacterial pan- and core genomes with PEPPAN.” *Genome Res* 30: 1667-1679. https://doi.org/10.1101/gr.260828.120

13. Katoh, Kazutaka, Kei-ichi Kuma, Hiroyuki Toh, Takashi Miyata. 2005. “MAFFT version 5: improvement in accuracy of multiple sequence alignment.” *Nucleic Acids Research* 33: 511-518. https://doi.org/10.1093/nar/gki198

14. Price, Morgan N., Paramvir S. Dehal, Adam P. Arkin. 2010. “FastTree 2--approximately maximum-likelihood trees for large alignments.” *PLoS One* 5: e9490. https://doi.org/10.1371/journal.pone.0009490

15. Gardner, Shea N., Tom Slezak, Barry G. Hall. 2015. “kSNP3.0: SNP detection and phylogenetic analysis of genomes without genome alignment or reference genome.” *Bioinformatics* 31: 2877-2878. https://doi.org/10.1093/bioinformatics/btv271

16. Marcais, Guillaume , Arthur L. Delcher, Adam M. Phillippy, Rachel Coston, Steven L. Salzberg, Aleksey Zimin. 2018. “MUMmer4: A fast and versatile genome alignment system.” *PLoS Comput Biol* 14: e1005944. https://doi.org/10.1371/journal.pcbi.1005944

17. Shi, Wenyu, Qinglan Sun, Guomei Fan, Sugawara Hideaki, Ohkuma Moriya, Takashi Itoh, Yuguang Zhou, et al. 2021. “gcType: a high-quality type strain genome database for microbial phylogenetic and functional research.” *Nucleic Acids Research* 49: D694-D705. https://doi.org/10.1093/nar/gkaa957

18. Zhang, Yifei, Dan Qiao, Wenyu Shi, Danni Wu, Man Cai. 2021. “Capnocytophaga periodontitidis sp. nov., isolated from subgingival plaque of periodontitis patient.” *Int J Syst Evol Microbiol* 71: https://doi.org/10.1099/ijsem.0.004979

19. Yu, Jie, Jie Zhao, Yuqin Song, Jiachao Zhang, Zhongjie Yu, Heping Zhang, Zhihong Sun. 2018. “Comparative Genomics of the Herbivore Gut Symbiont Lactobacillus reuteri Reveals Genetic Diversity and Lifestyle Adaptation.” *Front Microbiol* 9: 1151. https://doi.org/10.3389/fmicb.2018.01151

20. Xie, Fei, Wei Jin, Huazhe Si, Yuan Yuan, Ye Tao, Junhua Liu, Xiaoxu Wang, et al. 2021. “An integrated gene catalog and over 10,000 metagenome-assembled genomes from the gastrointestinal microbiome of ruminants.” *Microbiome* 9: 137. https://doi.org/10.1186/s40168-021-01078-x

21. Zhou, Zhemin, Nabil-Fareed Alikhan, Khaled Mohamed, Yulei Fan, Group Agama Study, Mark Achtman. 2020. “The EnteroBase user's guide, with case studies on Salmonella transmissions, Yersinia pestis phylogeny, and Escherichia core genomic diversity.” *Genome Res* 30: 138-152. https://doi.org/10.1101/gr.251678.119

# SUPPLEMENTARY FIGURES

**Figure S1.**

**Figure S2.**

**Figure S3.**

**Figure S4.**

**Figure S5.**

**Figure S6.**

**Figure S7.**
